# Zebrafish *brd2a *and *brd2b *are paralogous members of the bromodomain-ET (BET) family of transcriptional coregulators that show structural and expression divergence

**DOI:** 10.1186/1471-213X-8-39

**Published:** 2008-04-10

**Authors:** Angela J DiBenedetto, Jake B Guinto, Timothy D Ebert, Katharine J Bee, Michael M Schmidt, Todd R Jackman

**Affiliations:** 1 Department of Biology, Villanova University, Villanova, PA, USA; 2 Department of Neurology, University of Pennsylvania School of Medicine, Philadelphia, PA, USA; 3 Merck Pharmaceuticals, West Point, PA, USA; 4 Center for Molecular Cardiology, Weill Medical College of Cornell University, New York, NY, USA; 5 Department of Biological Engineering, Massachusetts Institute of Technology, Boston, MA, USA

## Abstract

**Background:**

Brd2 belongs to the bromodomain-extraterminal domain (BET) family of transcriptional co-regulators, and functions as a pivotal histone-directed recruitment scaffold in chromatin modification complexes affecting signal-dependent transcription. Brd2 facilitates expression of genes promoting proliferation and is implicated in apoptosis and in egg maturation and meiotic competence in mammals; it is also a susceptibility gene for juvenile myoclonic epilepsy (JME) in humans. The *brd2 *ortholog in *Drosophila *is a maternal effect, embryonic lethal gene that regulates several homeotic loci, including Ultrabithorax. Despite its importance, there are few systematic studies of *Brd2 *developmental expression in any organism. To help elucidate both conserved and novel gene functions, we cloned and characterized expression of *brd2 *cDNAs in zebrafish, a vertebrate system useful for genetic analysis of development and disease, and for study of the evolution of gene families and functional diversity in chordates.

**Results:**

We identify cDNAs representing two paralogous *brd2 *loci in zebrafish, *brd2a *on chromosome 19 and *brd2b *on chromosome 16. By sequence similarity, syntenic and phylogenetic analyses, we present evidence for structural divergence of *brd2 *after gene duplication in fishes. *brd2 *paralogs show potential for modular domain combinations, and exhibit distinct RNA expression patterns throughout development. RNA *in situ *hybridizations in oocytes and embryos implicate *brd2a *and *brd2b *as maternal effect genes involved in egg polarity and egg to embryo transition, and as zygotic genes important for development of the vertebrate nervous system and for morphogenesis and differentiation of the digestive tract. Patterns of *brd2 *developmental expression in zebrafish are consistent with its proposed role in *Homeobox *gene regulation.

**Conclusion:**

Expression profiles of zebrafish *brd2 *paralogs support a role in vertebrate developmental patterning and morphogenesis. Our study uncovers both maternal and zygotic contributions of *brd2*, the analysis of which may provide insight into the earliest events in vertebrate development, and the etiology of some forms of epilepsy, for which zebrafish is an important model. Knockdowns of *brd2 *paralogs in zebrafish may now test proposed function and interaction with homeotic loci in vertebrates, and help reveal the extent to which functional novelty or partitioning has occurred after gene duplication.

## Background

The BET family of proteins is defined by a dual bromodomain and an extra-terminal (ET) protein interaction domain [1]. The 110 amino acid bromodomain motif binds histones in an acetyl-lysine-dependent manner [2], and proteins carrying this module are major interpreters of the epigenetic histone code [3]. BET proteins in particular provide a scaffold for ordered recruitment, anchoring, and regulation of various chromatin modifying factors; they also act as adaptors that bridge sequence-specific transcription factors with the basal transcription machinery [4]. Ultimately, BET proteins regulate signal-dependent transcription as coactivators or corepressors, acting to maintain expressed or silenced states of gene expression [1,5], and have been implicated in meiosis [6], spermatogenesis [7], oocyte maturation [8], apoptosis [9], embryonic patterning [10] and especially, cell cycle control [11] and oncogenesis [12]. There is evidence for duplication and divergence of BET genes around the time of the emergence of vertebrates; accordingly, there are multiple members in each species, with four, *Brd2-5*, known in mammals [1].

The founding members of the Brd2 subfamily of BET proteins provide an evolutionary framework for functional analysis of this group. Yeast *bdf1 *encodes a basal transcription factor required for normal growth and meiosis [6,13]. The *Drosophila *homolog of *brd2*, *female sterile homeotic-1 *(*fsh1)*, is a maternal effect embryonic lethal gene required for oogenesis and proper segment formation and identity [10]. *fsh *interacts genetically with several homeotic genes, in some instances, via activation of *trithorax *genes (*trx-G*) [14]. Interestingly, *trx-G *genes encode various chromatin-interacting proteins and are antagonists of chromatin repressor *Polycomb *genes (*Pc-G*); together, the *trx-G *and *Pc-G *regulatory axes act to maintain proper *Homeobox *(*Hox*) expression patterns through development [15]. Brd2 is potentially a critical modulator of *Hox *cluster chromatin modification states through its effect on these major epigenetic axes [16]. Homologs of *trx-G *and *Pc-G *genes have antagonistic and homeotic regulatory functions in mice [17]; it remains to be seen if Brd2 is involved in *Hox *control in vertebrate development also. Surprisingly, *fsh *RNA and protein are distributed evenly in the embryo; spatial effects may result from restricted expression of co-regulators or downstream effectors [18].

The mammalian versions of *Brd2 *show both novel and overlapping functions compared with yeast and fly homologs. First, mouse/human Brd2 interacts with chromatin-modifying and transcription initiation complexes to affect gene transcription as part of pol II Mediator [11,19]. Specifically, Brd2 translocates to the nucleus upon mitogen-stimulation and enhances E2F-regulated gene transcription by recruiting cell cycle regulator E2F and a histone acetyl-transferase (HAT) to target promoters [11,19]. Mammalian Brd2 thus links cytoplasmic signal transduction to E2F-mediated entry into mitosis. Consistent with this, transgenic mice with lymphoid-restricted overexpression of Brd2 up-regulate E2F target gene cyclin A, and develop B cell lymphoma and leukemia [20]. Interestingly, several *trx-G *genes act as genetic regulators of E2F in *Drosophila *[21], and chromosomal abnormalities in the gene for *trx *human homolog *MLL*, underlie several forms of leukemia [22]; possibly, this reflects a conserved *Brd2/trx/E2F *regulatory pathway. Second, both mouse and human *Brd2 *genes produce testis-specific transcripts, and are implicated in spermatogenesis and/or oocyte maturation [7,8], thus maintaining a role in meiotic processes. In mouse oocytes,*Brd2 *RNA changes localization during oocyte maturation, from nuclear inclusion (immature) to nuclear exclusion (mature), perhaps increasing the fraction available for translation during meiotic reentry [8]. Whether this pattern holds true, and whether *Brd2 *acts as a maternal effect gene in vertebrates, is unknown. Third, both novel and conserved roles for *Brd2 *in development are likely. *Brd2 *RNAs are enriched in embryonic neuroepithelia of mouse brain ventricles, dorsal root ganglia, and spinal cord [23,24], and mouse Brd2 protein localizes to nuclei of proliferating neuronal precursors, but not of post-mitotic neurons [23], suggesting a role in vertebrate nervous system differentiation. *Brd2 *is also identified as a primary response gene transcriptionally induced in programmed cell death of rat neurons during development [9]. Recent studies corroborate a dual role for *Brd2 *in apoptosis and proliferation, showing Brd2 translocation to the nucleus of mouse mammary epithelial cells during both proliferative and post-lactation regressive stages [19], and demonstrating initiation of apoptosis in cultured cells by overexpression of Brd2 [25]. Consistent with a role in neuronal development, human *Brd2 *has recently been identified as the major susceptibility gene for juvenile myoclonic epilepsy [26]. A few single nucleotide polymorphisms (SNPs) in the *Brd2 *promoter may account for the phenotype, suggesting that regulated expression of this gene is critical for proper development of neural tissue [26].

Taken together, these studies implicate Brd2 as an important endpoint of signal transduction pathways regulating gene expression states necessary for proper differentiation, proliferation, and apoptosis during development and in adult tissues [27]. To identify and characterize both ancient conserved and novel derived functions, and to understand the structural and functional evolution of the Brd2 subfamily, parallel genetic and biochemical studies in different species are needed. To this end, we cloned *brd2 *cDNAs in zebrafish, analyzed sequence similarities and phylogeny with other known BET genes, and characterized overall RNA expression patterns during embryogenesis and in oocytes. In this report, we describe multiple *brd2*-related sequences in zebrafish, and present evidence for structural and expression divergence of *brd2 *paralogs in this species. The developmental profile of *brd2 *in zebrafish corroborates proposed functions from mouse and *Drosophila *studies, as well as suggests novel functions for the gene in vertebrate development.

## Results

### Cloning and sequence analysis of zebrafish *brd2*-related cDNAs

**Figure 1:**
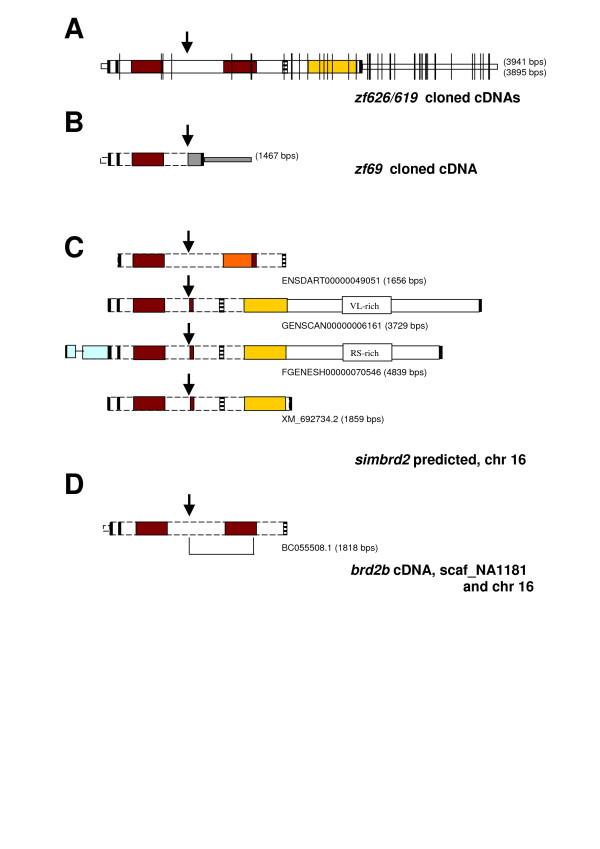
***brd2*-related genes encode predicted proteins with varying domain configurations**. Schematics of cloned and predicted *brd2*-related cDNAs with encoded protein domains are shown for A) cloned zf626 and zf619 cDNAs, B) cloned zf69 cDNA, C) supported and predicted *simbrd2 *transcripts from chromosome 16 ENSDARG00000046087 locus from Ensembl and NCBI databases, and D) *brd2b *partial cDNA from NCBI database. Some schematics include 5'UTR and 3' UTR, with wider rectangles depicting protein-coding regions. Boxes show conserved domains: bromodomain (red box); nuclear localization signal (horizontal striped); ET domain (yellow box); in C, degenerate second bromodomain (orange box); in C, upstream TAP domain (light blue box); valine-, leucine-rich region (VL-rich box); arginine-, serine-rich region (RS-rich box); start and stop codons (thick vertical lines); in A, SNPs between zf626 and zf619 (thin vertical lines); in B, discontinuous region in zf69 cDNA with repetitive sequences at bases 921–1033 and 1259–1413 (gray box); conserved intron/exon junction for alternative splicing in *brd2*-related transcripts (arrow). Two-exon region in *brd2b *cDNA found in scaf_NA1181 is underscored in D. Length of cDNAs in base pairs is given in parentheses.

We cloned the zebrafish homolog of *brd2 *by screening a 15–19 hour embryonic cDNA library ([28], see Methods). Schematics of three cDNA classes obtained, with encoded protein domains, are shown in Figure 1A and 1B. Two classes of cDNA, zf626 and zf619, are 98.6% identical, and encode predicted proteins of 836 and 838 amino acids, respectively (Fig. 1A). Proteins are identical except for an additional met/gly pair in zf619P, in a region of met/gly repeats, and contain features typical of the BET family [1], including dual bromodomains, a nuclear localization signal (NLS), and a C-terminal ET domain (Fig. 1A, patterned boxes). The zf626 and zf619 cDNAs differ from one another by several small deletions/additions, all but one of which occur in the 3'UTR, and a total of 44 SNPs (Fig. 1A, thin vertical lines). All 18 SNPs in the protein-coding region result in silent mutations, suggesting negative selection, while sequences in the 3'UTR show greater divergence (97.4% identity remains). The nature of these differences make it likely that zf626 and zf619 represent two independent, very closely related BET genes, or, divergent alleles of the same gene, rather than alternate transcripts. The third class of cDNA, zf69, encodes a predicted protein of 276 amino acids with a single bromodomain equivalent to bromodomain 1 of zf626/zf619, but lacking other BET features (compare Fig. 1A and 1B). The zf69 protein shares 70% identity with zf626/zf619 proteins across 276 amino acids of overlap, with 89.4% identity and 100% amino acid conservation across the bromodomain. At the level of nucleic acid, zf69 and zf626/zf619 show 63.3% and 79.5% identity, respectively, over these same regions. Thus, zf69 represents an independent bromodomain-containing gene, related to zf626/zf619, but atypical of the BET family. Starting at base 880, 48 bases upstream from the apparent stop of the zf69 open reading frame (Fig. 1B, vertical arrow), nucleotide similarity with zf626/zf619 falls abruptly to 43.1% (Fig. 1B, gray-filled portion). Blast searches identify repetitive sequences related to zebrafish Dana Sine and L3 Line repeat families in this discontinuous region, which was found to represent an alternative exon in zf69 cDNA (see below).

**Figure 9:**
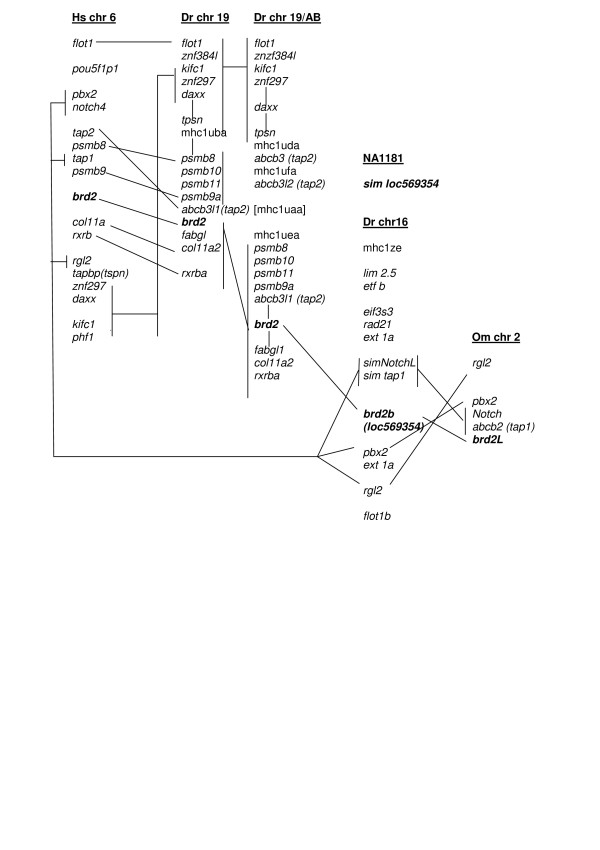
**Syntenic regions shared by zebrafish chromosomes 19 and 16, human chromosome 6, and trout chromosome 2**. Gene content of genomic regions where *brd2*-related sequences reside on human chromosome 6 (Hs chr 6), zebrafish chromosomes 19 (Dr chr 19) and 16 (Dr chr 16) from the Tubingen strain, zebrafish chromosome 19 from the AB strain (Dr chr 19/AB), and Onchorynchus chromosome 2 (Om chr 2). Gene order is derived from contig views in Vega (Dr chr 19, Dr chr 19/AB), Ensembl (Dr chr 16, Hs chr 6, NA1181), direct reading of annotated genomic clones (Dr chr 19, BX510994.6; Dr chr 19/AB, AL672176.10), and reference 28 (Om chr 2). Brd2-related genes are shown in bold. Genes that reside on the same genomic clone are shown in single-spaced groups; genomic clones within the same chromosome that physically overlap are connected by vertical lines. Related genes or groups of genes between chromosomes that show conserved synteny are also connected by lines. Human *brd2 *resides within the MHC II complex on chromosome 6, in a conserved syntenic block between *psmb8 *and *rxrb *genes; zebrafish *brd2 *is found in the same gene block within the fish MHC I core on chromosome 19, and is the true ortholog of mammalian *brd2*. Gene content on chromosome 19 from strains Tubingen and AB differs outside the *psmb8-rxrb *and the *flot1-tpsn *gene blocks, and may indicate recent genomic rearrangement. The zebrafish *brd2b *paralogous locus on chromosome 16 (alias loc 569354, *sim brd2*) is linked to *tap1 *sequences and other MHC II and III related genes, as is trout *brd2L *on chromosome 2, presumably representing the ancestral vertebrate configuration. *sim loc569354 *on unmapped scaffold NA1181 consists of two exons encoding the second bromodomain of *brd2b *partial cDNA (GenBank: BC055508), and with further genome assembly will likely be shown to derive from the same *brd2b *locus on chromosome 16.

To identify zebrafish sequences related to zf626/zf619 and zf69, we conducted similarity searches against NCBI, VEGA (version 24) and Ensembl (release 44) databases (see Methods). Several sequences show 99–100% identity to the cloned cDNAs (Table 1). zf626 and zf619 cDNAs derive from two distinct genomic clones, mapping to linkage group (LG) 19, that are predicted to encode zebrafish *brd2 *by structural homology and genomic synteny with the Major Histocompatibility Complex (MHC) (Table 1; see also Fig. 9). Surprisingly, the two genomic clones originate from different zebrafish strains: clone CH211-51F10 (encoding zf626) is from strain Tubingen (Table 1, BX510994), while clone BUSM-12F11 (encoding zf619) is from strain AB (Table 1, AL672176.10). At some point, mixing of Tubingen and AB genomes must have occurred, either in the construction of the cDNA library, or historically, via genetic introgression between strains. The only other known sequences corresponding to zf626/zf619 clones are two partial cDNAs with the zf619 configuration of SNPs (Table 1, BC045866.1 and AF032395.1), and sets of ESTs that exhibit either the zf626 or the zf619 SNP configuration (data not shown). Taken together, these data support the hypothesis that zf626 and zf619 represent divergent alleles of the *brd2 *gene on LG 19, marked by distinct strain-specific haplotypes. We obtained independent confirmation of the genomic location of zf626 and zf619 cDNA sequences by radiation hybrid mapping (see Methods).

**Table 1:** Danio sequences related to zf626, zf619 and zf69 cloned cDNAs

**cDNA**	**Database**	**Related zebrafish sequences In NCBI, Vega, Ensembl**	**LG**	**gene**	**BD1**	**BD2**	**NLS**	**ET**
**zf626**			19	brd2	x	x	x	x
	n,v,e	BX510994.6, genomic	19					
	n	XM_704349.2		brd2	x	x	x	x
	v	OTTDARG00000010014		brd2	x	x	x	x
	e	ENSDARG00000022280		brd2	x	x	x	x
								
**zf619**			19	brd2	x	x	x	x
	n,v,e	AL672176.10, genomic	19*					
	v	OTTDARG00000002248		brd2	x	x	x	x
	n	AL672164.13, 5'end	19*	brd2				
	n	BC045866.1, partial cDNA		brd2	x	x	x--?	
	n	AF032395.1, partial cDNA		brd2	p--?			
								
**zf69**			16	simbrd2	x	SINE		
	n	NW_001511437.1, genomic	16					
	n	XM_692734.2, loc569354		simbrd2	x	--	x	x
	e	Zv6scaffold 2405, genomic	16					
		ENSDARG00000046087		simbrd2/brd2b				
		ENSDART00000049051 (16.1)			x	p,d	x--?	
		GENSCAN00000006161 (16.2)			x	--	x	x
		FGENESH00000070546 (16.3)			x	--	x	x
	n	BC055508.1, partial cDNA	--	brd2b	x	x	x--?	
	n	NW_001514423.1, genomic	U					
		XM_001343674.1		simloc 569354	--	x/x--?		
	e	Zv6scaffold_NA1181, genomic	U					
		GENSCAN00000009613		simloc 569354	--	x/x/taa		
		FGENESCAN00000076064		simloc 569354	--	x/x-taa		

Sequences directly related to cloned zf626, zf619, and zf69 cDNAs were identified using blastn and blastp algorithms to search for similarities against both nucleotide and protein databases at NCBI (n), Vega version 24 (v), and zebrafish Ensembl release 44 (e). The linkage group (LG), gene name, and conserved domains are shown for each entry. *BD1*, bromodomain 1; *BD2*, bromodomain 2;*NLS*, nuclear localization signal; *ET*, extra terminal domain. Presence of conserved domains are indicated by "x". *p*, partial domain; *d*, degenerate domain;*-------?*, indicates truncation without stop codon;*-------taa*, indicates truncation with stop codon; *SINE/taa*, indicates repeat sequence with premature stop; 19*, genomic clones from chromosome 19 of strain AB. ENSDARG00000046087 locus contains loc569354, and potentially encodes four transcripts: one supported by evidence (*16.1*, ENSDART00000049051), and three predicted by different algorithms (*16.2*, GENSCAN00000006161; *16.3*, FGENESH00000070546; XM_692734.2).

We identified three sets of zebrafish sequences nearly identical to the region upstream of the discontinuous exon junction of zf69 cDNA: a predicted transcript (Table 1, XM_692734.2; Fig. 1C) from genomic contig NW_001511437.1 (locus 596354, *simbrd2*); a predicted transcript (Table 1, ENSDART00000049051; Fig. 1C) from Ensembl Zv6 scaffold2405 (locus ENSDARG00000046087); and a partial cDNA, *brd2b*, (Table 1, BC055508; Fig. 1C). Genomic contig NW_001511437.1 (NCBI) and Zv6 scaffold2405 (Ensembl) sequences are identical, and map to LG 16. We aligned genomic sequences with predicted and cloned transcripts, and found potential exons in locus ENSDARG00000046087 that correspond to each RNA, including the entire zf69. The discontinuous region begins at an intron/exon junction and is included as part of an exon in zf69; in XM_692734.2 and ENSDART00000049051, it is spliced as an intron. We confirmed that zf69 cDNA derives from ENSDARG00000046087 on chromosome 16 by radiation hybrid mapping (see Methods).

**Figure 2:**
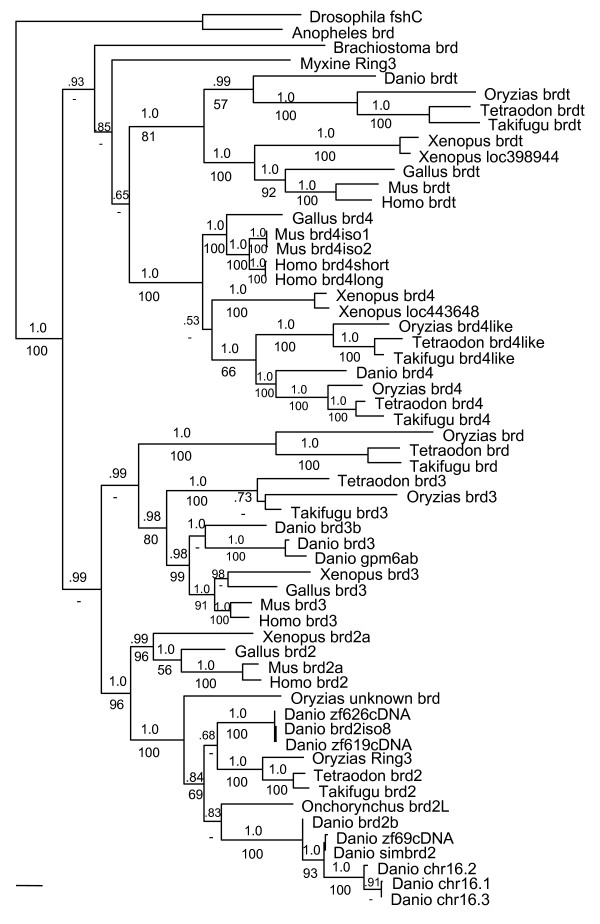
***brd2 *and *simbrd2/brd2b *represent a paralogous gene set in zebrafish**. Nucleic acid maximum likelihood tree of selected BET family homologs with Bayesian posterior probabilities (upper) and 1000× parsimony bootstrap values (lower) added to branches. At least one duplication of the *brd2 *gene has occurred in teleosts. zf626/zf619 cDNAs group with *brd2 *in zebrafish, while zf69 cDNA groups with sequences from the *simbrd2/brd2b *paralogous locus. *Danio *zf626, GenBank: EU126947; *Danio *zf619, GenBank: EU126946; *Danio *zf69, GenBank: EU126948; *Danio brd2iso8*, GenBank: XM_704349; *Danio simbrd2*, GenBank: XM_692734.2; *Danio *chr16.1, 16.2, 16.3, from Ensembl: ENSDARG00000046087; *Danio brd2b*, GenBank: BC055508; *Danio brd3*, GenBank: XM_693856; *Danio gpm6a*, GenBank: BC095027; *Danio brd3b*, GenBank: NM_212702; *Danio brd4*, GenBank: XM_694025; *Danio brdt*, GenBank: XM_701882; *Onchorynchus brd2L*, GenBank: ABB52829; *Takifugu brd2*, Ensembl: SINFRUG00000151665; *Takifugu brd3*, EMBL: AJ311635; *Takifugu brd4*, Ensembl: SINFRUG00000141925; *Takifugu brd4like*, Ensembl: SINFRUG00000145405; *Takifugu brdt*, Ensembl: SINFRUG00000148459; *Takifugu brd*, Ensembl: SINFRUG00000151665; *Tetraodon brd2*, EMBL: CAG11678; *Tetraodon brd3*, EMBL: CAF94980; *Tetraodon brd4*, EMBL: CAF92198; *Tetraodon brd4like*, EMBL: CAF90901; *Tetraodon brdt*, EMBL: CAF96012; *Tetraodon brd*, EMBL: CAF91369; *Oryzias Ring3*, GenBank: AB183488; *Oryzias brd3*, Ensembl: ENSORLG00000016659; *Oryzias brd4*, Ensembl: ENSORLG00000006490; *Oryzias brd4like*, Ensembl: ENSORLG00000010329; *Oryzias brdt*, Ensembl: ENSORLG00000013915; *Oryzias brd*, Ensembl: ENSORLG00000012190; *Oryzias Unknown brd*, Ensembl: ENSORLG00000016149; *Xenopus brd2*, GenBank BC043784; *Xenopus brd3*, EMBL: CAJ81450; *Xenopus brd4*, GenBank: BC076786; *Xenopus loc443648*, GenBank: BC097528; *Xenopus brdt*, EMBL: CAJ81450; *Xenopus loc398944*, EMBL: GenBank: BC060452; *Gallus brd2*, GenBank: NM_001030674; *Gallus brd3*, GenBank: XM_425330.1; *Gallus brd4*, Ensembl: ENSGALG00000013295; *Gallus brdt*, Ensembl: ENSGALG00000006031; *Mus Brd2*, GenBank: NM_010238; *Mus Brd3*, GenBank: NM_023336; *Mus Brd4iso1*, GenBank: NM_020508; *Mus Brd4iso2*, GenBank: NM_198094; *Mus Brdt*, GenBank: NM_054054; *Homo BRD2*, GenBank: NM_005104; *Homo BRD3*, GenBank: NM_007371; *Homo BRD4short*, GenBank: NM_014299; *Homo BRD4long*, GenBank: NM_058243;*Homo BRDT*, GenBank: NM_207189; *Myxine Ring3*, GenBank: AF191032; *Brachiostoma brd*, GenBank: AF391288; *Anopheles brd*, GenBank: XM_312107; *Drosophila fsh*, GenBank: NM_206647.

Interestingly, the ENSDARG00000046087 locus exhibits possible domain shuffling. The locus is predicted to encode two database-supported transcripts, and two model-derived *ab initio *transcripts (Table 1, XM_692734.2, ENSDART00000049051, GENSCAN00000006161, FGENESH00000070546, respectively), each of which is identical to zf69 from the start of the open reading frame to the intron/exon discontinuous junction (Fig. 1B,C vertical arrows), a region encoding the first bromodomain. At this point, all transcripts diverge, consistent with alternative splicing at this site, and predict proteins with different distal domains (Fig 1B,C patterned boxes): zf69 ends prematurely due to a stop codon in the included intron (Figs. 1B; 2, TAA); the ENSDART00000049051 protein has a degenerate second bromodomain and a nuclear localization signal; and GENSCAN00000006161, FGENESH00000070546, and XM_692734.2 each encode proteins with a distal fragment only of the second bromodomain followed by a nuclear localization signal, and the first two thirds of an ET domain. Other predicted features include C-terminal extensions of unknown function and an N-terminal transport-associated protein (TAP) domain (Fig. 1C). Finally, partial *brd2b *cDNA BC055508.1 encodes two canonical bromodomains, but ends prematurely without a stop codon at the NLS (Fig. 1D). NW_001511437/scaffold2405 genomic sequences account for all but two internal exons in BC055508.1 cDNA that encode the second canonical bromodomain. Using Blastn, we found these exons in an unmapped contig, Zv6_ scaffold NA1181, that shows at least 300 bases of overlap with NW_001511437. We believe further analysis will place NA1181 within NW_001511437, confirming the ENSDARG00000046087 locus on LG 16 as the source of all transcript isoforms, including *simbrd2 *and *brd2b*. Thus, several versions of Brd2-related proteins may exist, with different subsets of typical BET features, and with or without additional domains (Table 1).

### Sequence similarities and phylogeny of *brd2*-related sequences

Blastp searches of GenBank databases with zf626/zf619 and zf69 predicted proteins identify Brd2 orthologs as highest hits in other species. zf626/zf619 proteins share 72–73% identity with medaka [GenBank: BAD93258] and pufferfish [EMBL: CAG11678] Brd2, and 63–64% identity with human [GenBank: NP_005095] and mouse [GenBank: NP_034368] Brd2, over the entire sequence. In comparison, zf69 protein shows only 67–68% and 56% identity to other fish and mammalian Brd2 proteins, respectively, over 260 amino acids. All three predicted proteins are less similar to members of other BET subfamilies, namely Brd3, Brd4, and Brdt. Using ENSDART00000049051 predicted protein for comparisons gives similar results. Taken together, sequence similarity, domain architecture, and genomic location data verify zf626/zf619 as true zebrafish orthologs of *brd2*, and zf69 as belonging to the related *simbrd2*/*brd2b *locus, of uncertain position in the BET family.

To investigate the evolutionary relationship of these genes, we searched individual fish databases from *Oryzias*, *Tetraodon*, and *Takifugu *genome projects for similarities to zf626/zf619 and zf69. We identified all putative BET genes in fishes this way, and included them in phylogenetic analyses of BET vertebrate homologs (see Methods). Figure 2 shows a maximum likelihood tree with parsimony bootstrap and Bayesian posterior probability support values. For all trees, zf626/zf619 and zf69 group with other *brd2 *genes, rather than with BET paralogs (*brd3, brd4, or brdt*), and within the *brd2 *clade, zf626/zf619 groups with fish *brd2 *orthologs. zf69 and related *simbrd2/brd2b *sequences from chromosome 16 form a well-supported clade that may be seen as a subclade of the fish *brd2 *group, along with recently described *Onchorynchus *(trout) *brd2L *[29]. This, together with the *Oryzias *unknown *brd *sequence positioned just outside of the entire *brd2 *cluster in fishes, provide evidence for at least one duplication of ancestral *brd2 *in fishes, likely as part of the whole genome duplication thought to have occurred in teleosts after the split from the tetrapod lineage [30]. Subsequent gene loss in some fish species [30], or incomplete genomic data, may explain the failure to find *brd2b *orthologs in *Tetraodon *or *Takifugu*. Together, these data indicate that zf69/*simbrd2/brd2b *sequences represent a single *brd2 *paralogous locus on chromosome 16 in zebrafish. Following nomenclature guidelines for paralogs [31], this locus should be referred to as *brd2b *rather than *simbrd2*.

**Figure 3:**
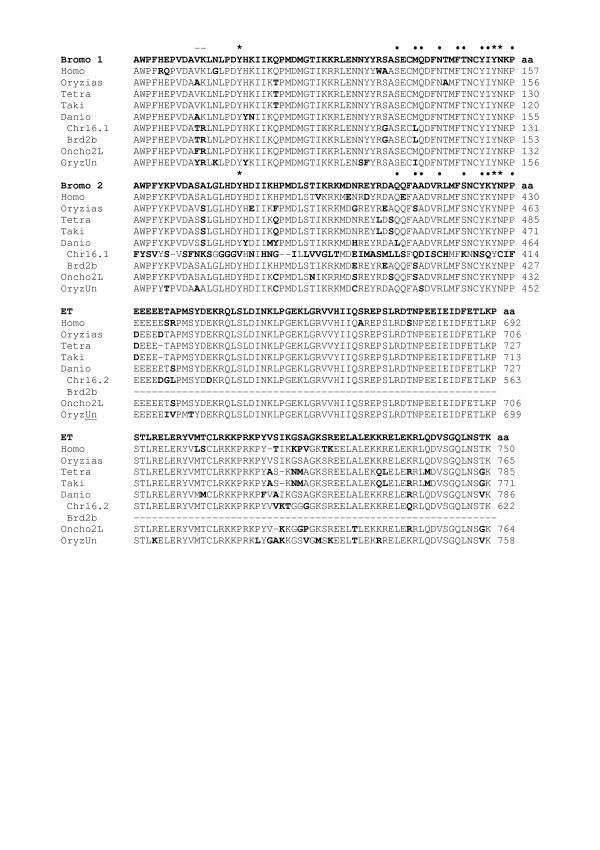
**Comparisons of core BET domains in selected Brd2 species homologs**. Amino acid sequences of core BET domains from Brd2 orthologs in Homo, Oryzias, Tetraodon, Takifugu, and Danio; and from Brd2 paralogous sequences in Danio (chr16.1 or chr16.2 for simBrd2; and Brd2b), Onchorynchus (Oncho2L for Brd2L) and Oryzias (OryzUn for Brd), are shown below consensus sequences (top line of each section, in bold), for bromodomain 1 (Bromo 1; 60 amino acid core), bromodomain 2 (Bromo 2; 60 amino acid core) and ET domain (ET; first 120 amino acids). Amino acid numbers at right show position relative to entire protein. Residues within domains that differ from consensus are shown in bold. Asteriks (*) denote bromodomain residues important for acetyl lysine-binding [3]. Dots (.) denote residues important for BD1/BD1 dimerization [32]. (--) shows varying residues in paralogs in ZA loop region. Danio is represented by zf626/Brd2 (Danio), and paralogous proteins encoded by ENSDART00000049051 (chr16.1, bromo 1 and 2), GENSCAN00000006161 (chr16.2, ET domain), and BC055508.1 cDNA (Brd2b; ends prematurely before ET domain). Sequences used here are translations of those used to construct phylogenetic trees (see Fig. 3 legend).

Figure 3 shows the alignment of core BET domains of zebrafish Brd2 and two chromosome 16 predicted proteins (16.1, ENSDART00000049051; 16.2 GENSCAN00000006161) with those of selected species homologs. As expected, the greatest similarity is found between domains of zebrafish Brd2 and other fish Brd2 proteins (eg. with Oryzias: bromo1, 98.3%; bromo2, 88.3%; ET, 95% identity), and similarity even to human Brd2 domains remains quite high (bromo1, 91.7%; bromo2, 83.3%; ET 85% identity). Domains of chromosome 16 paralogs are next highest in similarity to zebrafish Brd2 (16.1 bromo1, 95%; Brd2b bromo2, 87.7%; 16.2 ET, 89.2% identity), with the exception of degenerate bromodomain 2 of 16.1, which shows only 21.7% identity, although amino acid conservation remains high at 88.3%. Thus, in agreement with phylogenetic analysis in Fig. 2, Brd2 within-species paralogs are more divergent than between-species orthologs in fish, perhaps due to relaxation of selective constraints on conserved domain structure in paralogs. When compared with Onchorynchus Brd2L, chromosome 16 paralogs show higher similarity than does zebrafish Brd2, as expected, in bromodomains (Brd2 bromo1, 93%; 16.1 bromo1, 95%; Brd2 bromo2, 85%; Brd2b bromo2, 91.7% identity), but less similarity in ET domains (Brd2 ET, 94.1%; 16.2 ET, 92.4% identity); possibly, ET domains of paralogs have evolved to take on different interactions in zebrafish. Nevertheless, differences from consensus at identical positions among chromosome 16 protein paralogs and Onchorynchus Brd2L corroborate phylogenetic clustering of this group (Fig. 3, shared bold letters). Interestingly, two of these variant positions are found in the variable ZA loop of the predicted Brd2 bromodomain 1 (BD1) structure (Fig. 3, xx), which is thought to lend ligand selectivity to an otherwise highly conserved module [3]. Across Brd2 species homologs, BD1 and the first two thirds of the ET domain show the most amino acid identities. Recent structural work on Brd2 indicates BD1-BD1 homodimerization is critical for Brd2 function, placing strong selective constraints on the BD1-BD1 dimer interface region; these constraints compound those shared with bromodomain 2 (BD2) allowing heterodimer interaction (BD1-BD2) and acetyl-lysine binding [32]. In fact, residues identified as critical for BD1 dimerization and for acetyl-lysine recognition are 100% conserved in fish orthologs and paralogs (Fig. 3, dots). Besides BET domains, most Brd2 homologs have pre-NLS PEST motifs (poly-glu, poly-ser) indicating rapid turnover, and terminal SEED motifs (poly-ser, with glu, asp) of unknown function [1] (see additional file 1). A significant difference among vertebrate Brd2 proteins concerns the configuration of putative non-canonical ser/thr kinase motifs [12,33]. Although consensus sequences for kinase subdomains (YHRDLK and APE) within bromodomain 2, and for catalytic glutamate (EKR) within the ET domain, are present in fish, bird and mammalian Brd2, a potential ATP-binding motif with catalytic lysine (GxGxxG and AxK) [33] in the characteristic position after the NLS, is found only in bird and mammalian proteins; in *Danio *and *Oryzias*, motifs do not exhibit conserved spacing. Chromosome 16 paralogs lack kinase motifs of any kind.

### Developmental profile of *brd2 *and *simbrd2/brd2b *genes

**Figure 4:**
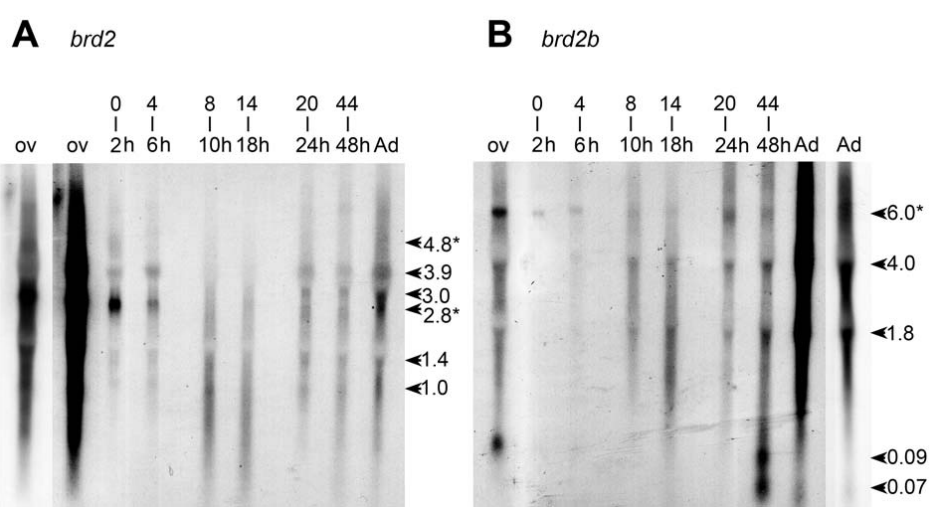
***brd2a *and *brd2b *encode gonad-enriched RNAs and multiple developmentally regulated transcripts**. Northern blot of total RNA (5 μg/lane) from dissected ovary (ov), adult fish (Ad), and embryos of various stages (0–2, 4–6, 8–10, 14–18, 20–24, and 44–48 hour post fertilization) probed sequentially with A) zf626 (*brd2*) and B) zf69 (*brd2b*), radiolabeled cDNA subfragments. RNAs were visualized with EtBr staining and were undegraded and of equivalent amounts in each lane (data not shown). The leftmost ovary lane and the rightmost adult lane are underexposed replicates of adjacent lanes, to allow better band visualization. Ovary-enriched *brd2 *RNAs (A, 4.8 and 2.8 kb) and ovary-enriched *brd2b *RNA (B, 6.0 kb) are also present in early embryos (A and B, 0–2 h), and are indicated by asterisks (*). Various size classes of *brd2 *and *brd2b *RNAs present in whole adults are also detectable in dissected testis (data not shown).

Little is known about the expression of *brd2 *in vertebrate development, except for a few intriguing studies in mouse [19,20]. We therefore examined RNA patterns for *brd2a *during zebrafish embryogenesis, and for comparison, sampled the expression of closely related paralog *brd2b*. We generated probes specific for zf626/zf619 *(brd2a*, chr 19) and for zf69 *(brd2b*, chr 16); probes could not distinguish between different transcript isoforms from the same locus however (see Methods). Figure 4 shows Northern blots of *brd2a *and *brd2b *expression during development, and in dissected ovaries and adult fish. Each probe detects multiple regulated transcripts and, as in other *brd2 *orthologs, one or more gonad-enriched RNAs. Two classes of *brd2a *RNAs are highly enriched in ovaries and 0–4-hour post-fertilization (hpf) embryos (Fig. 4A, 4.8 and 2.8 kb). Interestingly,*brd2a *transcripts appear degraded in 8–18 hour embryos, even though RNA in these lanes is intact by EtBr staining (data not shown), and the same lanes show intact transcripts when probed with zf69 (compare same lanes in part B). Possibly, a change specifically in *brd2a *turnover rate occurs during segmentation. Three size classes of *brd2b *RNAs are detected in fish from 8 hpf. to adult (Fig. 4B, 6.0 kb, 4.0 kb, 1.8 kb); the largest is enriched in ovaries and 0–2 hour embryos. Intriguingly, small *brd2b*-related transcripts of 70–90 bases are apparent in pharyngula stage embryos (44–48 hpf) and ovaries. Overall, sizes of detected *brd2*a and *brd2b *RNAs could potentially accommodate predicted transcripts for these genes (Fig. 1).

**Figure 5:**
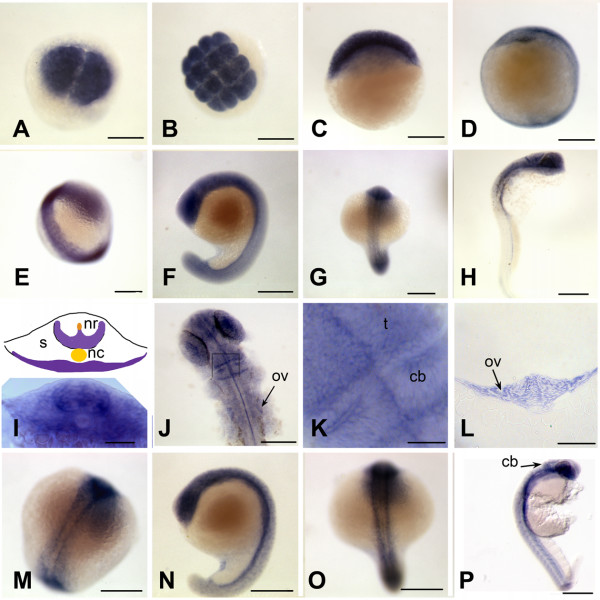
***brd2 *paralogs are enriched in developing nervous system during segmentation**. Whole mount *in situ *hybridizations to RNA in zebrafish embryos conducted with DIG-labelled zf626 (*brd2*) (A-L) and zf69 (*brd2b*) (M-P) cloned sequences. Ubiquitous expression is seen in embryos of 2 cell (A), 16 cell (B), 30% epiboly (C), and bud (D) stages for both *brd2 *(shown) and *brd2b *(not shown) genes. Enriched expression in "ladder" patterns along the developing neural keel/rod during segmentation is seen in 14 somite embryos probed for *brd2 *(E) and for *brd2b *(M). Cross-sections of 14 somite whole mount embryos probed for *brd2 *(I, schematic with real image below; 5–6 somite level) show enriched expression in ventrolateral neural rod (nr), floorplate above notochord (nc), and ventral somites (s). Expression is most prominent in head region of 18 somite embyros for both paralogs (F, G; and N, O); only *brd2 *is also enriched in tail bud (F), while *brd2b *remains high along the entire ventral trunk (N) at this stage. By 24 hours, *brd2 *is prominent in the entire head, with some expression in ventral trunk and post-anal tail (H), while *brd2b *is enriched in ventral brain, cerebellum, and ventral trunk, with low level ubiquitous expression (P). Dorsal flat mounts of 24 hour embryos (J, K) show *brd2 *expression enriched within caudal tectum (t) at hindbrain-midbrain boundary, within specific cells of the cerebellum (cb) and at the caudal border between cerebellum and rest of hindbrain. The image in (K) is a higher magnification view of a region from another embryo equivalent to the boxed region in J. Expression of *brd2 *in cells of otic vesicle walls (ov) is apparent both in flat mounts (J) and cross sections at that level (L). Bar = 250 μm for A-H, J, M-P; = 50 μm for I, K; = 100 μm for L.

**Figure 6:**
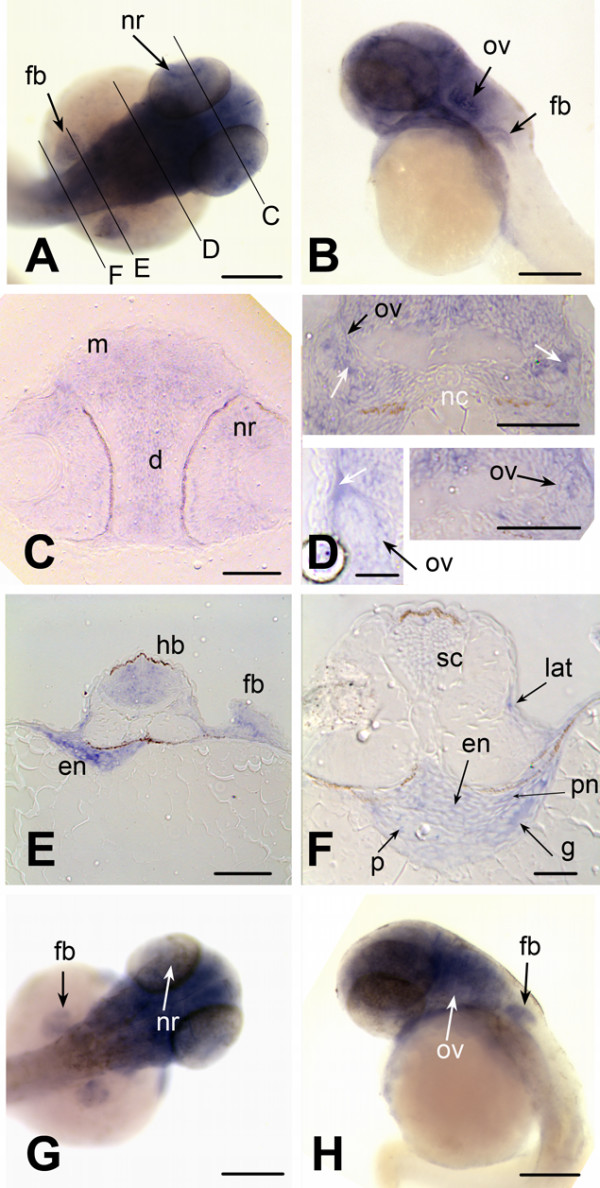
***brd2 *paralogs are differentially expressed in head and trunk regions of pharyngula stage embryos**. Whole mount *in situ *hybridizations to RNA in 44–48 hour zebrafish embryos with DIG-labelled zf626 (A-F) and zf69 (G, H) cloned sequences. Dorsal (A) and lateral (B) views of whole embyros show *brd2 *expression abundant in all brain subdivisions and developing pharyngeal arches, and in neural retina (nr), otic vesicle (ov), pectoral fin buds (fb) and ventral trunk. Cross sections at levels indicated in A, reveal *brd2 *expression in: C) mesencephalon (m), diencephalon (d) and neural retina (nr); D) in cells of otic vesicle walls (ov, black arrows); E) in caudal hindbrain (hb), endoderm of prospective digestive tract (en), and pectoral fin bud (fb); F) in developing spinal cord (sc), posterior lateral line primordium (lat), pronephric duct (pn), and endodermal tissue (en), including pancreas progenitor (p) and developing gut (g). Signal in hollow of otic vesicle (white arrows in D) is an artefact due to trapped probe. Although *brd2b *(G dorsal, H lateral) is also expressed in head region and pectoral fin buds (fb), it is reduced in dorsal midbrain, caudal hindbrain, and ventral trunk, and absent from neural retina (nr, white arrow) and otic vesicle (ov, white arrow). Bar = 250 μm for A,B,G,H; = 100 μm for C, D (two right images); = 25 μm for D (left image).

**Figure 7:**
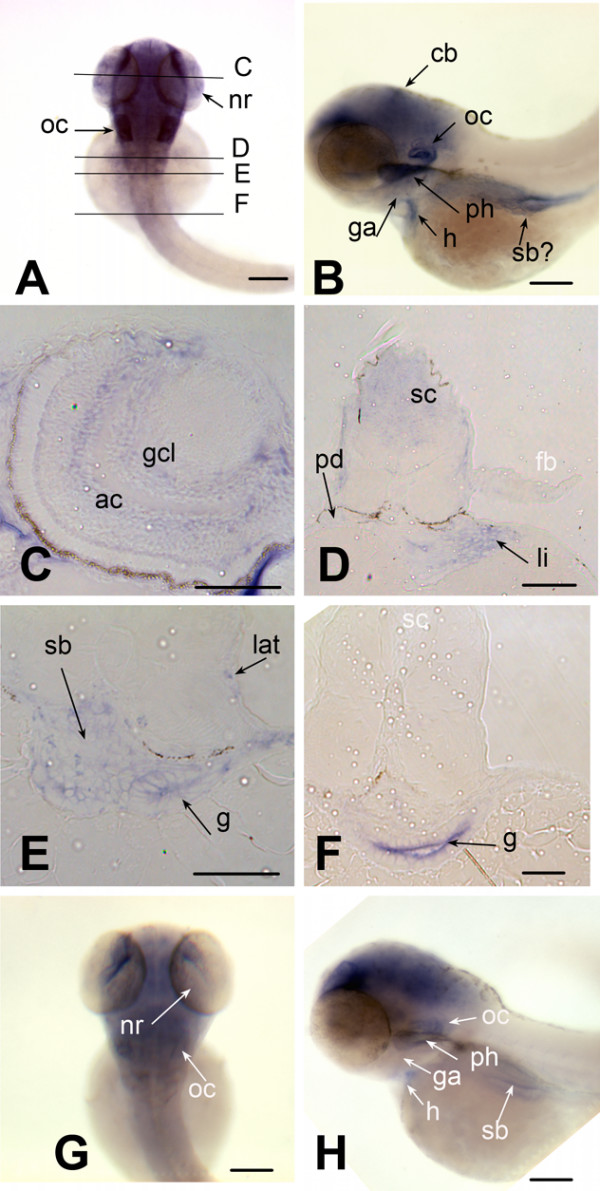
***brd2 *paralogs are differentially expressed in head and digestive system of pecfin stage embryos**. Whole mount *in situ *hybridizations to RNA in 60–65 hour zebrafish embryos with DIG-labelled zf626 (A-F) and zf69 (G, H) cloned sequences. Dorsal (A) and lateral (B) views of whole embryos show *brd2 *expression in all brain subdivisions, especially cerebellum (cb), in neural retina (nr), otic capsule (oc), atrium of heart (h), gill arches (ga), pharynx (ph), swim bladder (sb) and ventral trunk. Cross sections at levels indicated in A reveal *brd2 *expression in: C) amacrine cells (ac) and ganglion cell layer (gcl) of neural retina; D) spinal cord (sc), pronephric duct (pd) and endodermal derivatives such as liver (li); E) posterior lateral line primordium (lat), and endodermal derivatives such as swim bladder and gut (g). *brd2 *expression continues in gut (g), but declines in spinal cord (sc, white) as more posterior sections are assayed (F), and is severely reduced in fin bud (D; fb, white) Expression of *brd2b *(G dorsal, H lateral) is found mainly in head region, but is reduced overall, especially in hindbrain, and nearly absent from otic capsule (oc, white), gill arches (ga, white), heart (h, white), ventral trunk, and endodermal derivatives such as pharanx (ph, white), and swim bladder (sb, white). Bar = 250 μm for A,B,D,G,H; = 100 μm for C,E,F.

**Figure 8:**
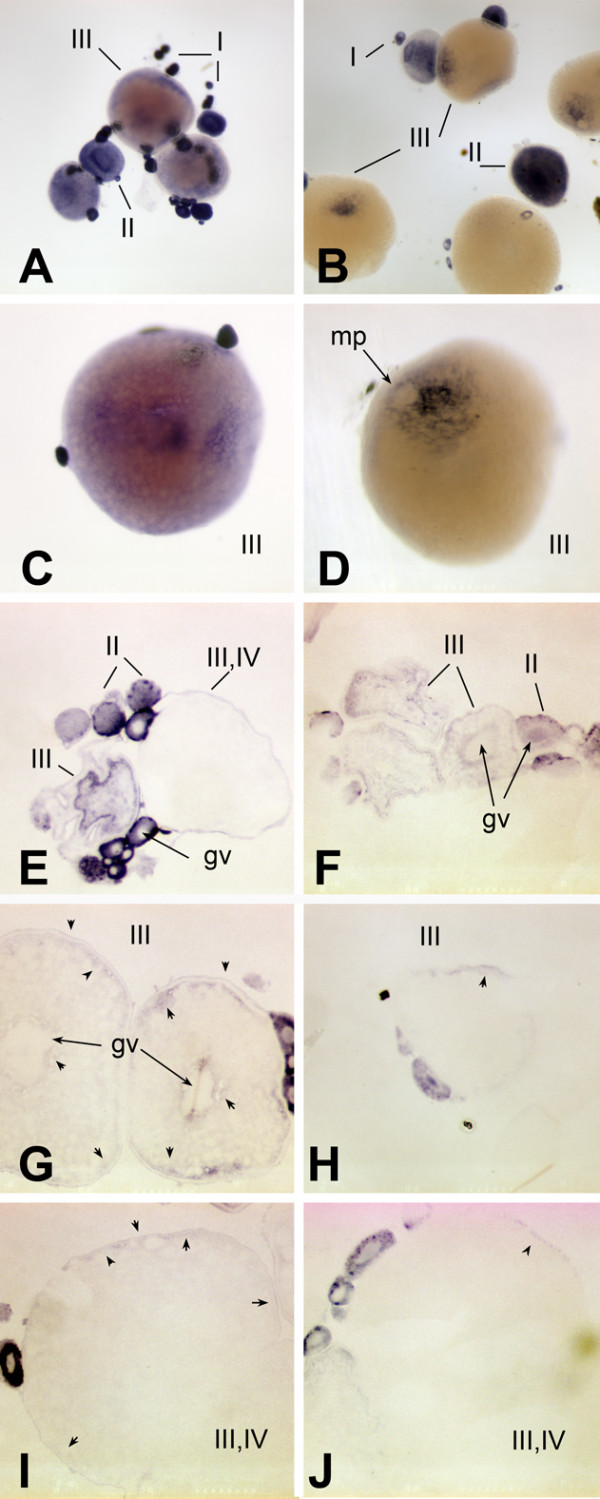
***brd2 *paralogs are maternal transcripts with different patterns of expression in oocytes**. Whole mount *in situ *hybridizations to RNA in zebrafish ovaries with DIG-labelled zf626 (A, C, E, G, I) and zf69 (B, D, F, H, J) cloned sequences. Cross sections through ovaries are shown in E-J. *brd2 *maternal RNAs are dispersed and abundant in stage I and II oocytes (A, E), and are sometimes found inside the germinal vesicle (gv), as in G (see small oocytes at far right). By stage III,*brd2 *transcripts accumulate around the outside of the germinal vesicle and rather unevenly around the periphery of the cortex just inside the vitelline envelope (C, E; G, arrowheads). *brd2 *RNAs then localize around the periphery of the cortex by late stage III, early stage IV (I, arrowheads). *brd2 *RNAs are also present in follicle cells of stage III/IV oocytes (C; G, I, arrowheads outside oocyte). Although *brd2b *maternal transcripts are also dispersed throughout stage I and II oocytes, and often found inside the germinal vesicle (B, F, H, J), in contrast to *brd2*, they become strictly localized to the region surrounding the micropyle (D, mp; H, J, arrowheads), the future animal pole, sometime during stage III, and are not expressed in follicle cells. A,B: 40X; C-F: 80X; G-J, 100X.

To visualize expression of *brd2a *and *brd2b *in zebrafish embryos, the same probes were used for hybridization to RNA *in situ *(Figs. 5, 6, 7, 8). Both *brd2a *and *brd2b *RNAs are present at high levels in the earliest embryos (Fig. 5A–C), consistent with their being maternally-supplied transcripts. They become enriched in optic primordia and developing brain and nervous system during segmentation (Fig. 5E–H;M–P). In early through mid-segmentation, as sensory, motor and interneurons appear and neural keel is forming neural rod [34,35], both sets of RNAs exhibit a periodic, ladder-like pattern of enrichment longitudinally along the trunk anterior-posterior (A-P) axis, visible over lower level ubiquitous expression (Fig. 5E,G;M,O). Ladder patterns differ in thickness and relative intensity over time between the two genes (Fig. 5 compare E and M; G and O). Cross-sections through 14-somite stage embryos show ladder patterns arise in part due to strongly enriched expression in ventral somites and in ventral-lateral neural keel/rod and floorplate, which in fact give rise to motor and interneurons (Fig. 5I; [35]). The 18 somite embryo shows *brd2a *concentrated in the 10 brain neuromeres and tailbud and generally reduced in trunk, while *brd2b *remains high in brain and along the ventral trunk, but is relatively reduced in tailbud (Fig. 5F versus 5N). At this stage both genes are expressed in otic placode (data not shown) and optic primordia (Fig. 5F,N). Thus, early expression of *brd2a *and *brd2b *implicate these genes in formation of the vertebrate nervous system, in agreement with studies in mice [23]. By 24 hours (prim 5) expression of the two paralogs begins to differ significantly. *brd2a *is now restricted to head, eye, otic vesicle, neural tube, developing pectoral fin buds, ventral trunk, and dorsal post-anal tail (Fig. 5H,J). At higher magnification,*brd2a *appears enriched in neural retina and cells of otic vesicle walls (Fig. 5J,L), at midbrain/hindbrain boundary and specific regions of the cerebellar primordium (Fig. 5J,K), and in anteriorly migrating blood island cells from ventral trunk (data not shown). Expression of *brd2b *at 24 hours is much less restricted; highest levels are seen in cerebellum, optic primordia and ventral trunk (Fig 5P). By 48 hpf, both genes are expressed in developing heart, pharyngeal arches, pectoral fin buds, and brain, but *brd2b *is absent from neural retina, and reduced in otic vescicle, dorsal midbrain, and caudal hindbrain compared to *brd2a *(Fig. 6A and 6B versus 6G and 6H). Cross sections of 48 hour embryos show additional *brd2a *expression in posterior lateral line primordia, pronephric ducts, and in endodermal tissues that give rise to gut, pancreas, liver, and swim bladder (Fig. 6C–F). At this stage, *brd2a *expression in neural tube is much reduced (Fig. 6F). At 60 hours (pecfin stage), whole mounts show *brd2a *persistent in all brain subdivisions, pharyngeal arches, neural retina, otic capsule, and ventral trunk in the region of developing endoderm derivatives and pronephric duct; it is now absent from pectoral fins, and shows *de novo *expression in developing pharynx, esophagus, swim bladder, and atrium of the heart (Fig. 7A,B). In comparison, *brd2b *expression in head is reduced in caudal hindbrain and otic capsule, and is absent from neural retina, pharyngeal arches, heart, and ventral trunk (Fig. 7G,H). Cross sections of 60 hour embryos confirm *brd2a *expression in brain, epithelial lining of pharyngeal arches, neural retina, endodermal derivatives, pronephric duct, and lateral line primordia (Fig. 7C–F). Finally, both paralogs are expressed exclusively in developing swim bladder and midbrain in 5 day fish (see additional file 2). Thus, later developmental expression of *brd2 *paralogs provides evidence for functional divergence between these genes, and implicates *brd2a *in patterning and morphogenesis of brain, eye, ear, pectoral fins and digestive tract and associated organs, before terminal differentiation.

Since both *brd2a *and *brd2b *appeared to be maternal transcripts, we examined gene expression in unfertilized oocytes. We found strikingly different patterns for the two genes (Fig. 8). *brd2a *RNA is abundant and dispersed throughout the oocyte cytoplasm in early stages I and II, and is sometimes seen within the germinal vesicle (Fig. 8A,E). It starts to accumulate rather unevenly around the cortical circumference and around the outside of the germinal vesicle in early stage III oocytes; RNA is also present in surrounding follicle cells (Fig. 8A,C,E;G, arrows inside and outside oocyte). By late stage III-stage IV,*brd2a *RNA is mainly limited to two thin domains on either side of the vitelline membrane: one, at the cortical circumference of the oocyte, the other, in the surrounding follicle cells (Fig. 8I, arrows inside and outside oocyte). Like *brd2a*, *brd2b *RNA is at first dispersed throughout the cytoplasm of stage I and II oocytes, and sometimes seen enriched within the germinal vesicle (Fig. 8B,F). It also accumulates near the cortical circumference and outside the germinal vesicle in early stage III (Fig. 8F). Later in stage III, however, *brd2b *RNA becomes strictly localized within the cortical region around the micropyle, a specialized follicle cell that marks the future animal pole (Fig. 8D;E,J, arrows). Thus, both *brd2 *paralogs are implicated as vertebrate maternal effect genes, with potential roles in egg polarity and egg to embryo transition; in addition, their strikingly different localization patterns again support divergence in function.

## Discussion

### Brd2 structure and evolution

We describe two sets of *brd2 *paralogous sequences in zebrafish, located on chromosomes 19 and 16, and present evidence for their structural and expression divergence. Syntenic relationships between these genes and their homologs support our phylogenetic analysis. The two genomic clones from which zf626 and zf619 strain-specific cDNAs derive map within the zebrafish MHC class I core locus on chromosome 19, which exhibits conserved synteny with the MHC region on human chromosome 6 containing *brd2 *[36], verifying zf626 and zf619 as true *brd2 *orthologs (Fig. 9, compare Hs chr 6 with Dr chr 19 and Dr chr 19/AB). Interestingly, differences in gene content outside two syntenic blocks (*psmb8*-*rxrb*, and *flot1-tpsn*) on chromosome 19, between Tubingen (Dr chr 19) and AB (Dr chr 19/AB) zebrafish strains, provide evidence for recent rearrangement, associating blocks with either *mhcI uba *or *mhcI uda/ufa/uea *genes, respectively (Fig. 9, non-italic genes). Similarly, a recent intrachromosomal duplication of MHC I core region in salmonids has resulted in shuffling of *mhcI *genes flanking *psmb-rsrb *blocks in trout [29]. In fact, the history of MHC loci across vertebrate species is one of frequent rearrangement, presumably due in part to a preponderance of retrotransposons [37]; nevertheless, these same gene blocks retain conserved synteny in fish and mammals [30,38]. As *brd2a *resides in such a block (Fig. 9, *psmb8-rxrb*), its conserved position within the MHC is likely functionally significant (see below). zf69 and related transcripts from the *brd2b *locus on chromosome 16 group phylogenetically with *brd2L *from *Onchorynchus *chromosome 2. As expected, zebrafish chromosome 16 and trout chromosome 2 share synteny with eachother, and both share a number of MHC-linked genes with zebrafish chromosome 19 and with human chromosome 6 (Fig. 9, compare Dr chr 16, Om chr 2, Dr chr 19, Hs chr 6). In fact, the linkage of *brd2b *with MHC class II and III genes (*tap1, pbx2, notch-L*), as seen in the paralogous loci, is hypothesized to reflect the ancestral vertebrate MHC configuration, while the linkage of *brd2a *with MHC class I core genes on zebrafish chromosome 19 and trout genomic contig [GenBank: DQ139863] is considered derived – the result of teleost-specific duplication, translocation and/or inversion events [29].

*brd2 *paralogs in zebrafish show potential for domain-shuffling at the identical phase 0 exon junction, suggesting that gene duplication is not merely redundant, but has allowed functional diversification of *brd2 *to occur in fishes. For instance, *brd2b *isoform zf69 encodes a single bromodomain (equivalent to BD1) protein lacking an ET domain. Since BD1-BD1 homodimers can form and bind acetylated histone tails [32], zf69P dimers might act as dominant negative molecules, binding to chromatin as a cargo-less adaptors. In fact, all chromosome 16 isoforms encode a canonical BD1, but only one also encodes a canonical BD2; three have no BD2 and one has a degenerate BD2. This suggests a scenario whereby BD1 homodimerization could bring together various combinations of Brd2b isoforms, with and without BD2, ET or terminal extensions. In addition, Brd2b proteins may show differences in ligand selectivity compared with Brd2a, due to paralog-specific amino acid substitutions in the variable ZA loop of BD1 [3]. Thus, the Brd2b group possibly reads different acetyl-lysine signals of the histone code [3], and/or targets different promoters, in addition to being expressed differentially, compared to Brd2a. Cross species comparison shows the C-terminal third of ET to be the least conserved domain in the Brd2 subfamily, suggesting that varying affinities for interacting proteins might add to functional diversity. Finally, only chicken, mouse and human Brd2 have the expected configuration of kinase motifs, and the existence of intrinsic kinase function is still controversial [1]. Indeed, recent work indicates recruitment rather than kinase activity is critical for Brd2 nuclear translocation and transactivation functions [20]. Contig views at Vega and Ensembl show the presence of multiple repetitive elements in *brd2 *loci across species, especially in fish (see additional file 1). In fact, the chromosome 16 locus has SINE/LINE elements in conserved intronic positions, where alternate splicing may produce multiple *brd2b*-related isoforms. Possibly, these sequences allow higher frequencies of rearrangement tending to the modular nature and structural diversity of *brd2 *paralogs in zebrafish [37]. Cloning and functional testing of these proposed isoforms and their interacting partners may provide unique insights into mechanisms of gene diversification after duplication [39].

### *brd2 *paralog expression and potential function in oocytes and embryos

We propose that *brd2a *and *brd2b *act as maternal effect genes in vertebrates, with a role in oogenesis and egg to embryo transition. As in *Drosophila*, mouse and zebrafish *brd2 *RNAs are present in oocytes and early embryos [8,10,23]; but unlike *fsh *transcripts, the RNAs show changing patterns of localization throughout oogenesis [8], suggesting vertebrate *brd2 *may act by different proximal mechanisms. Mouse and zebrafish *brd2a*, but not zebrafish *brd2b *or *Drosophila fsh*, are also expressed in surrounding follicle cells [8,10,18], reflecting perhaps a novel vertebrate function in somatic ovary for *brd2a*. Location of both mouse and zebrafish RNAs changes from diffuse cellular in early oocytes to nuclear exclusion in mature oocytes; in mouse, this final shift is correlated with the ability of oocytes to re-enter meiosis [8]. Unique to zebrafish, cytoplasmic RNAs from both *brd2 *paralogs become further localized in stage III oocytes, supporting involvement in egg polarity. Given that Brd2 acts as a major histone-directed transcriptional co-regulator and recruitment scaffold [1,3,5], it may potentially play a role here in epigenetic reprogramming of the oocyte and/or activation of the zygotic genome in the egg to embryo transition [40]. The localization of *brd2a *and *brd2b *into cortical and animal pole classes [41], respectively, could thus reflect partitioning of related genes into separate and reciprocal regulatory domains at this critical juncture [40]. In addition, the fact that recruitment and translation of maternal RNAs needed for zygotic transcriptional activation may be coordinated with cell cycle progression [40], suggests that the proposed *brd2/trx/E2F *epigenetic pathway inferred from work on Brd2 in mammals and *Drosophila *[11,15,17,19,21], might be operative here (see Introduction).

Expression patterns of *brd2 *paralogs in zebrafish embryos support the idea of a *brd2/trx/Hox *pathway in vertebrate development. First, *brd2 *RNAs in zebrafish, and *trx, Ultrabithorax (Ubx)*, and *Antennapedia (Antp) *RNAs in flies, show similar profiles: enrichment in the central nervous system of the later embryo superimposed upon lower ubiquitous levels [42]. Second, "ladder patterns" of zebrafish *brd2 *RNAs appear during somitogenesis and persist while fine-grained A-P segmental patterns of motoneurons are being established by differential *homeobox *expression, and interneuron axons are extending along longitudinal tracts within the embryonic spinal cord [34,35]. Consistent with this, *brd2a *RNA is enriched in ventrolateral neural keel/rod, where motorneurons and interneurons originate. Third, our *in situ *hybridizations also show *brd2a *expression appearing in order in zebrafish gut and associated organs as they develop along the A-P axis, with foregut derivatives showing expression last [43]. Interestingly, both cephalic homeosis and midgut disruptions are seen in *Drosophila *carrying the *fsh *allele *ranc*or, with loss of zygotic function that ordinarily regulates *homeobox *genes such as *labial *and *Deformed *[44]. In a role analogous to *fsh *in *Drosophila *patterning and segment identity [1,10,14,18], zebrafish *brd2 *paralogs might regulate *homeobox *genes via *trithorax*-related functions in a canonical pathway for vertebrate central nervous system and/or gut patterning and morphogenesis [35,43]. Two recent findings support this idea: first, *Drosophila *FSH acts directly at the *Ubx *promoter and synergistically activates transcription together with *trxG*-encoded proteins residing at distal enhancers [45], confirming at the molecular level genetic interactions observed previously [10,14,18,44]; and second, E2F regulator and tumor-suppressor pRB, cooperates with a *trithorax *gene product, RB-binding protein 2 (RBP2), to activate the Brd2 promoter in differentiating mammalian cells, suggesting that Brd2-dependent maintenance of active chromatin states and/or modulation of *Homeobox *gene expression are effectors of RB-mediated differentiation [16]. Intriguingly, then, *brd2/trx/E2F *and *brd2/trx/Hox *pathways might intersect via master regulator pRB, a protein known to control cell fate decisions at the crossroads of death, differentiation and division [46]. For instance, *brd2/trx/E2F *might facilitate cell cycle progression in the absence, and *brd2/trx/Hox *promote differentiation in the presence, of active RB [16]. Apropos of this, expression of mouse and zebrafish *brd2 *in specific precursors and primordia during times of ongoing morphogenesis and differentiation, followed by reduction upon terminal differentiation [23], could also reflect *brd2 *modulation of cell death and proliferation [9,19,23,25], which together help shape tissues and organs during morphogenesis.

The conserved linkage of *brd2 *in the vertebrate MHC – as the lone gene not directly involved in antigen presentation [47] – makes some sense in light of the pathways mentioned above. First, constitutive lymphoid expression of Brd2 results in B cell lymphoma [20] characterized in part by transactivation of proliferation-promoting genes, including E2F-regulated cell cycle genes [48]. Second, *Hox *gene expression is dynamically modulated in adult hematopoietic lineages, where differentiation is ongoing, and *Hox *gene dysregulation underlies various malignant leukemias, including those associated with genetic rearrangements of *trithorax *homolog *MLL *[49]. Thus, the *brd2/trx *epigenetic axis could be pivotal to maintenance of appropriate expression states of both *Hox *and cell cycle genes throughout hematopoiesis, and thus to proper function of the adult immune system [49].

Finally, gene expression patterns implicate both mouse and zebrafish *brd2 *in continued development of brain. Consistent with this, structural defects in various brain regions in JME patients suggest an underlying abnormal neural network [50,51], with differences in *Brd2 *expression during development suspected factors due to promoter SNPs that correlate with disease [26]. Whether misregulation of *Brd2 *results in inappropriate mitosis or cell death, or in deregulation of *homeobox *genes, is unknown. The function of *Brd2 *during development and its connection with JME can now be analyzed by gene knockdown and ectopic expression studies in zebrafish, which is an important model for human epilepsy [52]

## Conclusion

Zebrafish *brd2 *gene paralogs shows potential for functional diversity based on differences in structural domain configurations and distinct RNA expression patterns in oocytes and developing embryos. The developmental expression pattern of zebrafish *brd2 *corroborates findings from studies in fly, mouse and human, and provides new evidence for *brd2 *function as a maternal effect and zygotic gene in vertebrates, with potential roles in oogenesis, egg to embryo transition, and proper development of the digestive and central nervous systems. Brd2 paralogs provide unique opportunities for distinguishing the roles of maternal and zygotic genomes in constructing the vertebrate embryo, for investigating interactions with important developmental regulators, and for analyzing the extent to which divergence after gene duplication consists in functional novelty or functional partitioning.

## Methods

### Cloning of zebrafish *brd2*-related cDNAs

Gene-specific primers were designed from a 330 base partial clone of zebrafish *Ring3 *[GenBank: AF032385] [28] and used to amplify a probe from the *brd2 *gene using zebrafish genomic DNA as template (5' primer: ATTATTACCGCAGTGCCAGC; 3' primer: TTGTTTTCCTCTGGGGACAG). Genomic DNA was isolated from adult zebrafish by standard protocol [53]. PCR was conducted using 1 μg genomic DNA, 0.5 μg of each primer, 0.2 mM dNTPs, and 2.5 U Taq polymerase (Promega), for 30 cycles: 92°C for 1.5 min, 40°C for 1 min, 70°C for 1 min. PCR product was cloned into TA vector (Invitrogen), and verified by sequencing. [^32^P]-dCTP-radiolabeled probe from cloned insert was synthesized using Prime It kit (Stratagene) and used to screen a 15–19 hour zebrafish (strain AB) embryonic cDNA library in lambda ZAP (gift of B. Appel, University of Oregon) by standard plaque hybridization at 42°C in hybridization buffer containing 50% formamide [54]. 25 positive clones and ten independent isolates were obtained after 3 rounds of rescreening.

### Sequence and phylogenetic analysis of cDNA clones

Both strands of cloned cDNA were sequenced by cycle sequencing as per company protocol (PE Biosystems) using an ABI 373A automated sequencer. Open reading frames were analyzed using DNA Strider 1.3 [55], predicted protein features, using bioinformatics tools at ExPasy [56], and similarities to known genes, using blastn, blastx and blastp programs on databases at NCBI [57], Vega version 24 [58], and Ensembl release 44 [59], and on databases from genome projects of individual species including Oryzias [60], Tetraodon [61], and Takifugu [62]. Sequence comparisons and initial alignments among *brd2 *clones and homologs were performed using ClustalW [63] and MegAlign [64]. Phylogenetic trees were created by first aligning amino acid sequences using ClustalW and adjusting alignments by hand in MacClade [65], followed by analysis of corresponding nucleotide sequences using PAUP*4.0 [66]. Trees were created using Bayesian analysis with posterior probabilities [67], and reiterated 1000 times to obtain parsimony bootstrap values. Syntenic regions on zebrafish chromosomes 19, 16 and 9 and human chromosome 6 were derived from contig views using Vega, NCBI and Ensembl platforms, or directly from NCBI and Vega annotated genomic clones. Map positions of cloned zf626, zf619, and zf69 cDNAs were obtained by sending 3' UTR sequences to the zebrafish radiation hybrid mapping service at the Zon lab Genomics site [68].

### Northern blot analysis

Total RNA was extracted from zebrafish adults, dissected testis and ovaries, and embryos at various time points post fertilization (0–2 h, 4–6 h, 8–10 h, 16–18 h, 24 h, 48 h, and 72 h p.f.), using TriReagent according to manufacturer's protocol (Molecular Research Products). 2 μg of each sample was fractionated in 1% agarose, 0.66 M formaldehyde gels in 1× MOPS and transferred to nylon filters (Hybond) in 10× SSPE buffer. Gene-specific probes were generated from gel-purified (GeneClean kit II, Bio 101) subfragments unique to each cDNA, that excluded highly conserved or repetitive regions. A PCR-generated fragment (base 631-base 1231 of clone *zf626*) from the region between bromodomains was subcloned into pGemT (Promega) for *brd2a*-specific probes; an EcoR1 fragment containing the first 862 base pairs of clone *zf69*, that excludes SINE/LINE family repetitive sequences, was subcloned into pBluescript (Stratagene) for *brd2b*-specific probes. Hybridizations in buffer (0.5 M phosphate, pH 7, 7% SDS, 1% BSA, 20 mM EDTA, pH 8) containing 1 × 10^6 ^cpm/ml of [^32^P]-dCTP-labeled insert were conducted at 55°C overnight. Filters were washed twice in 2× SSPE/2% SDS at room temperature, and twice in 0.2× SSPE/0.2% SDS at 55°C, before being exposed to Xray film.

### *In situ *hybridization to mRNA in embryos and ovaries

*In situ *hybridizations to RNA in whole mount zebrafish embryos were conducted according to the Schulte-Merker protocol as found in the Zebrafish Book [53], with slight modification. Acetic anhydride and proteinase K treatment were used on older embryos only (>18 hpf). Digoxigenin (DIG) labeled RNA probes were prepared according to Roche Biochemical instructions, and used directly in hybridizations without prior hydrolysis. Hybridizations were conducted with 1 ng/ml probe in 50% formamide buffer with 5 mg/ml torula yeast (type VI, Sigma), and without heparin, at 65°C overnight. Post-hybridization washes (50% formamide, 2× SSCT; 2× SSCT; 0.2× SSCT) were carried out at 55°C, without RNase treatment. Embryos were blocked in PBST plus 10% FBS, 0.1% Tween-20, and 1% DMSO for 4 hours before anti-DIG detection in staining buffer plus levamisol. After detection, embryos were fixed overnight in 4% paraformaldehyde, then dehydrated and stored in methanol at -20°C. For photography, embryos were cleared in 2 parts benzylbenzoate: 1 part benzylalcohol and mounted in Canada balsalm. For *in situ *to ovaries, the high resolution whole-mount protocol of Thisse as described in The Zebrafish Book [53], was followed exactly.

## Authors' contributions

ADB conceived the study, led in its design and coordination, performed final sequence and syntenic analyses and *in situ *hybridizations, and drafted the manuscript. JBG carried out the library screens and initial categorization of clones. TDE carried out initial *in situ *hybridizations and expression analysis. KJB carried out initial sequence analysis and phylogenetics, and conducted detailed comparison of cDNA clones. MMS analyzed genomic sequences, ESTs, and sequence similarities of *brd2*-related genes in zebrafish using bioinformatics. TRJ performed phylogenetic analyses.

## Supplementary Material

Additional file 1 **Structural domain comparison among Brd2 species orthologs**. Domain comparisons among Brd2 species orthologs, including the zebrafish Brd2 paralogous set from LG 19 and 16, and Brd2 from other teleosts, xenopus, chicken, mouse and human. *B1*, bromodomain 1; *B2*, bromodomain 2; *Kin*, kinase domain; *Pest/NLS*, poly glutamic acid, poly serine motif/nuclear localization signal; *ATP/catK/catE*, ATP-binding domain with catalytic lysine or glutamic acid; *ET/SEED*, extra-terminal domain/poly serine, with glutamic acid, aspartate; *Other*, non-BET domain. Presence of domains is indicated by "x". *p*, position is not conserved; *disc*, sequence discontinuity due to alternative splicing; *SINE/taa*, included intron with repetitive elements and stop codon; *deg*, degenerate sequence; *-----?*, truncation without stop codon; *TAP*, transport-associated protein domain; *VL*, valine-, leucine-rich region; *RS*, arginine-, serine-rich region. The location of repetitive SINE/LINE sequences within introns is indicated by numbered flanking exons, or within upstream or downstream flanking regions, by (5') or (3'), respectively. *, equivalent exon/exon junction. See Figure 3 legend for sequence accession numbers. Click here for file

Additional file 2 ***brd2 *paralogs are expressed in swim bladder and brain in 5 day old zebrafish**. Whole mount *in situ *hybridizations to RNA in 5 day old zebrafish with DIG-labelled zf626 (A) and zf69 (B) cloned sequences. Lateral views show *brd2a *and *brd2b *expression restricted to swim bladder (sb), otic capsule (oc) and brain, with *brd2b *RNAs most abundant in swim bladder. Bar = 250 μm. Click here for file
