# Supplementary material for: Zebrafish brd2a and brd2b are paralogous members of the bromodomain-ET (BET) family of transcriptional coregulators that show structural and expression divergence
Source: BMC Dev Biol. 2008 Apr 10;8:39. doi: 10.1186/1471-213X-8-39 (PMC2373290; doi:10.1186/1471-213X-8-39)
Supplement: Additional file 1 — Structural domain comparison among Brd2 species orthologs. Domain comparisons among Brd2 species orthologs, including the zebrafish Brd2 paralogous set from LG 19 and 16, and Brd2 from other teleosts, xenopus, chicken, mouse and human. B1, bromodomain 1; B2, bromodomain 2; Kin, kinase domain; Pest/NLS, poly glutamic acid, poly serine motif/nuclear localization signal; ATP/catK/catE, ATP-binding domain with catalytic lysine or glutamic acid; ET/SEED, extra-terminal domain/poly serine, with glutamic acid, aspartate; Other, non-BET domain. Presence of domains is indicated by "x". p, position is not conserved; disc, sequence discontinuity due to alternative splicing; SINE/taa, included intron with repetitive elements and stop codon; deg, degenerate sequence; -----?, truncation without stop codon; TAP, transport-associated protein domain; VL, valine-, leucine-rich region; RS, arginine-, serine-rich region. The location of repetitive SINE/LINE sequences within introns is indicated by numbered flanking exons, or within upstream or downstream flanking regions, by (5') or (3'), respectively. *, equivalent exon/exon junction. See Figure 3 legend for sequence accession numbers. [file 1471-213X-8-39-S1.doc]

**Additional file 1-Table 2- Structural domain comparison among Brd2 species orthologs**

| **Transcript** | **BD1** | **BD2** | **Kin** | **Pest/NLS** | **ATP/catK/E** | **ET/SEED** | **other** | **SINE/LINE** |
| --- | --- | --- | --- | --- | --- | --- | --- | --- |
| Brd2 **Danio** | x | x | x | x/x | p/p/x | x/x |  | 8/9; 11/12 |
| Zf69 cDNA | x | disc | SINE/taa |  |  |  |  | 6/7* |
| Chr16.1 | x | deg | -- | x/x---? |  |  |  | 5; 2/3; 4/5*  6/7; 10/11; 3’ |
| Chr16.2 | x | disc | -- | x/x | -/-/- | x/- | VL | multiple |
| Chr16.3 | x | disc | -- | x/x | -/-/- | x/- | TAP; RS | multiple |
| Simbrd2 | x | disc | -- | x/x | -/-/- | x/x |  | 6/7* |
| Brd2b | x | x | x | x/x---? |  |  |  |  |
| Brd2 **Oryzias** | x | x | x | x/x | p/p/x | x/x |  | Other rep |
| Brd2 **Tetra** | x | x | x | x/x | -/p/x | x/x |  | Other rep |
| Brd2 **Taki** | x | x | x | x/x | -/p/x | x/x |  | Other rep |
| Brd2 **Xeno** | x | x | x | x/x---? |  |  |  | Other rep |
| Brd2 **Gallus** | x | x | x | x/x | x/x/x | x/x |  | 10/11; 3’ |
| Brd2 **Mus** | x | x | x | x/x | x/#/x | x/x |  | 10/11; 3’ |
| Brd2 **Homo** | x | x | x | x/x | x/x/x | x/x |  | 10/11; 3’ |

Domain comparisons among Brd2 species orthologs, including zebrafish Brd2 paralogs. *BD1*, bromodomain 1; *BD2*, bromodomain 2; *Kin*, kinase domain; *Pest/NLS*, poly glutamic acid, poly serine motif/nuclear localization signal; *ATP/catKtE*, ATP-binding domain with catalytic lysine or glutamic acid; *ET/SEED*, extra-terminal domain/poly serine, with glutamic acid, aspartate; *Other*, non-BET domain. Presence of domains is indicated by “x”. *p*, position is not conserved; *disc*, sequence discontinuity due to alternative splicing; *SINE/taa*, included intron with repetitive elements and stop codon; *deg*, degenerate sequence; *-----?*, truncation without stop codon; *TAP*, transport-associated protein; *VL*, valine-, leucine-rich region; *RS*, arginine-, serine-rich region. Location of repetitive SINE/LINE sequences within introns is indicated by numbered exons, or within flanking regions, by (5’) or (3’). *, equivalent exon/exon junction. See Figure 2 legend for sequence accession numbers.
