# Supplementary figures and images for: Zebrafish brd2a and brd2b are paralogous members of the bromodomain-ET (BET) family of transcriptional coregulators that show structural and expression divergence
Source: BMC Dev Biol. 2008 Apr 10;8:39. doi: 10.1186/1471-213X-8-39 (PMC2373290; doi:10.1186/1471-213X-8-39)

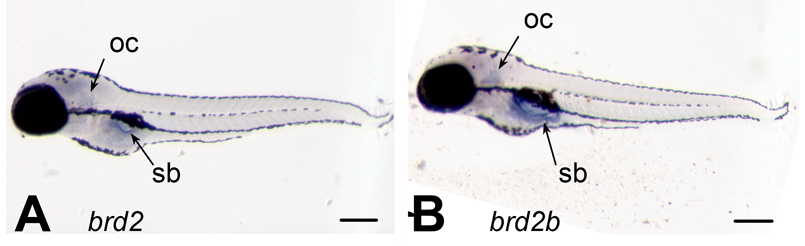

Supplement: Additional file 2 — brd2 paralogs are expressed in swim bladder and brain in 5 day old zebrafish. Whole mount in situ hybridizations to RNA in 5 day old zebrafish with DIG-labelled zf626 (A) and zf69 (B) cloned sequences. Lateral views show brd2a and brd2b expression restricted to swim bladder (sb), otic capsule (oc) and brain, with brd2b RNAs most abundant in swim bladder. Bar = 250 μm. [file 1471-213X-8-39-S2.png]
